# Midterm results of Ilizarov hip reconstruction for late sequelae of childhood septic arthritis

**DOI:** 10.1007/s11751-014-0202-2

**Published:** 2014-10-11

**Authors:** Mahmoud A. El-Rosasy, Mostafa A. Ayoub

**Affiliations:** Department of Orthopaedic Surgery, Faculty of Medicine, Tanta University Hospital, University of Tanta, Al-Geish Street, Tanta, Egypt

**Keywords:** Hip joint, Septic arthritis, Ilizarov, Pelvic support osteotomy

## Abstract

The management of hip instability as a consequence of septic arthritis in childhood is difficult. Ilizarov hip reconstruction is a double-level femoral osteotomy with the objective of eliminating hip instability, through a proximal valgus–extension–derotation osteotomy and a distal varization–lengthening osteotomy for mechanical axis correction and equalization limb length. Ilizarov hip reconstruction was performed for 16 adult patients with complaints of hip pain, leg-length discrepancy, limping, reduced activity and limited abduction of the hip as a result of childhood septic arthritis. Their ages ranged from 19 to 32 years (mean 23.2 ± 4.2). Ilizarov external fixator was used in all cases. At the time of last follow-up that ranged from 60 to 132 months (mean 85.6 ± 23.5), the Harris hip score (HHS) showed excellent functional outcome in two cases (12.50 %), good in 13 cases (81.25 %) and fair in one case (6.25 %). There was no poor functional outcome in any case. Preoperatively, the mean HHS was 56.18 points, and at the time of last follow-up, it improved to a mean of 84.62 points. Pain subsided in all patients, the Trendelenburg sign became negative in all but three (19 %) patients, no patient had limb-length discrepancy, and the alignment of the extremity was reestablished in all cases. No additional operations were required. Ilizarov hip reconstruction is a valuable and durable solution for the late sequelae of childhood septic arthritis of the hip presenting in adult patients.

## Introduction

The late sequela of septic arthritis of the hip in childhood is a complex problem. The symptoms are a limp, with or without pain, limb-length discrepancy, deformity and hip stiffness. The management of this problem in young adults is controversial. Arthrodesis of the hip joint offers a stable, painless but immobile hip joint, which makes sitting on a chair and in public transportation difficult and can render difficulties with perineal hygiene. Moreover, conversion of a fused hip into total hip arthroplasty is challenging and has some higher risks, and this is not the ideal choice for young patients due to the increased activity and the need for frequent revisions [1–5].

The pelvic support osteotomy (PSO) was described to solve the problem of hip instability by transferring body weight to the femoral shaft through a proximal valgus osteotomy [6]. Although this osteotomy can provide sufficient pelvic support, it results in lateralization of the limb and did not address limb-length discrepancy [7]. Ilizarov added a second, more distal varus femoral osteotomy for lengthening to restore the overall alignment of the lower limb [8].

This study presents our midterm (minimum 5-year follow-up) results of the Ilizarov hip reconstruction (IHR) procedure using the Ilizarov external fixator in adult patients who have a unilateral hip disorder after septic arthritis of the hip.

## Materials and methods

Sixteen adult patients constitute the cohort of this retrospective study. The ages range from 19 to 32 years (mean 23.2 ± 4.2 years). There are nine males and seven female patients. The inclusion criteria were a unilateral hip insufficiency after septic arthritis of the hip in childhood and with a minimum postoperative follow-up of 5 years.

**Fig. 1:**
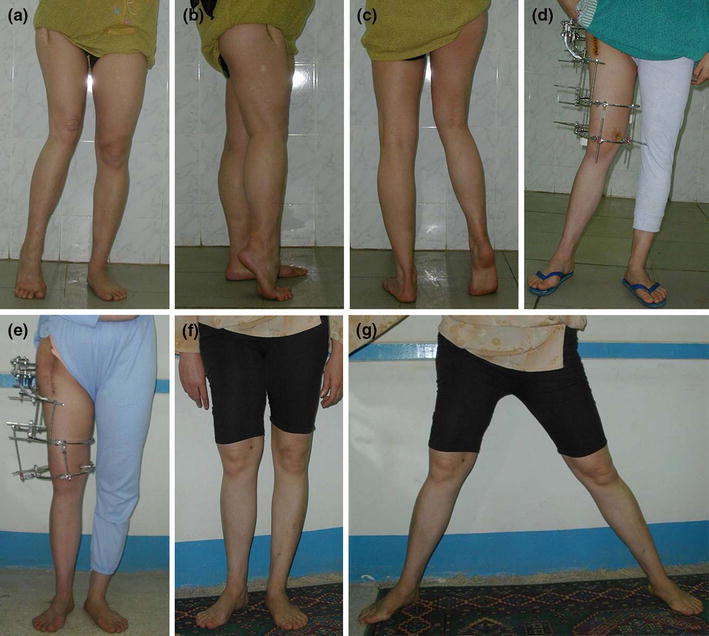
**a**–**c** Preoperative clinical photographs of a 32-year-old female patient with sequelae of septic arthritis in childhood affecting the right hip joint. The patient had flexion, adduction, internal rotation deformities and an apparent limb-length discrepancy of 12 cm. **d** Postoperative photograph shows the applied Ilizarov frame, valgus of the lower limb due to the proximal osteotomy and reduction in the limb-length discrepancy to 4 cm. **e** Photograph after 4 cm lengthening and correction limb alignment by varus angulation at the distal osteotomy. **f**, **g** Follow-up photographs show the functional outcome

The presenting symptoms were a limp (with or without pain), leg-length discrepancy, deformity, hip stiffness and, in some, difficulty with marital relationship (Fig. 1a). Surgery in all cases was performed by the first author and data collection and evaluation at final follow-up by the second author who was not involved in surgery.

### Preoperative evaluation

This included a general assessment of the medical status and patient’s expectations from the treatment. Local examination was done to record the presence of pain, scars of previous surgery, sinuses, fixed pelvic obliquity and fixed contractures of the hip, limb-length discrepancy (by the block method) and the neurological, muscle strength and vascular status of the lower limb.

Plain radiographs were obtained for the lumbosacral spine (to detect fixed scoliotic deformity), pelvis and hip joints. A CT scan of the pelvis and both hip joints was obtained for better evaluation of the hip joint regarding the location of the femoral head relative to the acetabulum and presence of fibrous or bony ankylosis of the joint.

### Procedure

The procedure was performed with the patient in the supine position. An adductor tenotomy was performed through a medial approach. Mobilization of the damaged hip was performed by undoing the ankylosis through a limited anterolateral approach to the hip and under image intensifier, where the remnants of the femoral head were removed piecemeal using a bone rongeur. The affected limb was then maximally adducted over the other limb so that the upper part of the femur was seen parallel to the lateral wall of the pelvis on the affected side and abutting against the ischium; that point of abutment was marked as the site of the proximal osteotomy. With the limb maximally adducted, half pins were inserted in the proximal femur perpendicular to the sagittal plane of the patient’s body and parallel to the horizontal plane. A fixation block consisting of one small and one large arch from the Ilizarov system was attached to these pins. The limb was then abducted back to the neutral position, and a preconstructed Ilizarov frame was then applied to the femur distal to the proposed proximal osteotomy with the pins inserted perpendicular to the axis of the distal femur. The proximal osteotomy was performed percutaneously using a drilling and osteotome method. Holding both the proximal block and the distal part of the frame, the osteotomy was manipulated to affect a derotation–extension–valgus osteotomy but with care to avoid excessive displacement and loss of bone contact. The proximal and distal parts of the frame were connected using threaded rods and oblique supports. The level of the distal osteotomy was determined preoperatively so that the mechanical axis of the lower limb passes through the acetabulum and the knee joint line of the knee remains horizontal. The distal osteotomy was performed percutaneously.

### Postoperative management and follow-up

**Fig. 2:**
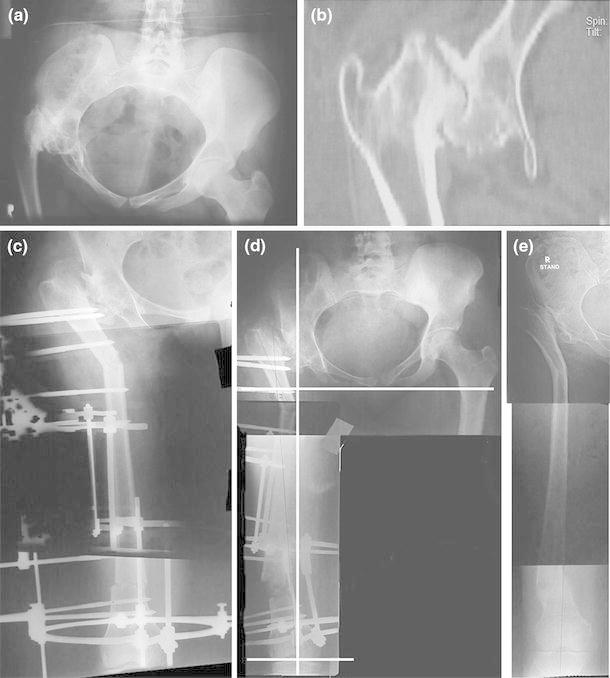
**a**, **b** Preoperative plain radiograph and CT scan show destruction of the femoral head and acetabulum and fibrous ankylosis as a result of septic arthritis of the right hip in childhood. The radiograph shows evidence of hip adduction, flexion (non-visualized obturator foramen) and internal rotation (non-visualized lesser trochanter). **c**, **d** Radiographs during treatment show the proximal and distal osteotomies, lengthening and varus through the distal osteotomy. The knee joint-orientation line is horizontal and proximal extension of the mechanical axis of the distal femur passes through the acetabulum. **e** Follow-up radiograph shows the consolidated osteotomies and maintained alignment

The patient was mobilized after postoperative pain had subsided. The patient was encouraged to mobilize the lower limb joints and bear weight as tolerated. The amount of limb lengthening needed was estimated using the block method; in the assessment, an awareness of the fixed pelvic obliquity was maintained so as not to overestimate the actual discrepancy (there is an adduction contracture present frequently). Distraction of the distal osteotomy was started after 5–7 days at a rate of 1 mm per day until the limb-length discrepancy was corrected. Thereafter, hinges were built into the external fixator to gradually move the distal segment into varus until the knee joint line was horizontal (Fig. 1b–d). After consolidation of the osteotomies, the frame was removed without anesthesia (as an outpatient procedure usually). No cast was applied. Figure 2 demonstrates the procedure radiologically and the follow-up result.

### Evaluation of results

The results were recorded from the patients’ notes. Clinical and radiological assessments and subsequent data analysis and calculation of scores were performed at the time of last follow-up by the second author. The functional outcome was graded using the Harris hip score (HHS). This has a total of 100 points: Pain receives 44 points; function, 47 points (activities of daily living have 14 points and gait 33 points); range of motion, 5 points; and deformity, 4 points. A total score below 70 points is considered a poor result; 70–79, a fair result; 80–89, a good result; and 90–100, an excellent result [9].

The follow-up radiographs were assessed for orientation of the knee joint line and restoration of the overall mechanical axis of the lower limb, such that extension of the mechanical axis of the tibia and distal femur should pass through the acetabulum with a horizontal knee joint-orientation line.

Statistical analysis was done using SPSS version 11.0.1 for Windows (SPSS Inc., Chicago, IL, USA). The one-sample *t* test was used for dependent variable mean comparison, and one-way analysis of variance (ANOVA) test and its nonparametric equivalent, the Kruskal–Wallis test, were used for variables that were small and not normally distributed. A *p* value ≤0.05 was considered to be statistically significant.

## Results

There were 16 patients with ages ranging from 19 to 32 years (mean 23 ± 4.2 years). There were nine (56 %) male and seven (44 %) female patients. The right side was affected in nine (56 %) cases and the left side in seven (44 %) cases. A fixed flexion deformity of the hip was present in all cases and ranged from 20 to 45° (mean 32.8 ± 7.9°). A fixed adduction contracture of the hip was present in eleven cases (69 %) and ranged from 10 to 25° (mean 16.8 ± 5.1°). A fixed internal rotation deformity was present in eight cases (50 %) and ranged from 5 to 15° (mean 10.6 ± 3.2°). Preoperative limb-length discrepancy was present in all cases and ranged from 3 to 12 cm (mean 5.6 ± 2.3 cm); after release of the ankylosis and fixed contractures, this shortening when reassessed (postoperatively and prior to the start of lengthening) was found to have a mean 2.8 (as measured clinically by the block method, Table 1). The surgical release of the ankylosis of the hip joint improved the apparent preoperative LLD significantly (*p* ≤ 0.001).

**Table 1:** Preoperative demographic data of the patients

No.	Age (years)	Sex	Side	Fixed hip deformities			LLD (cm)	
				Flexion	Adduction	Internal rotation	Preoperative	Postoperative
1	19	Male	Left	40°	15°	10°	8	2.5
2	32	Female	Right	45°	25°	15°	12	4
3	20	Male	Left	30°	20°	0°	6	3
4	19	Male	Left	20°	15°	0°	4	2
5	22	Male	Right	35°	10°	0°	5	2
6	21	Female	Right	40°	15°	10°	7	4
7	24	Female	Right	25°	0°	5°	4	2.5
8	30	Male	Left	30°	0°	0°	4	2
9	19	Male	Left	30°	20°	0°	3	2
10	20	Female	Right	35°	15°	10°	5	3
11	24	Female	Right	45°	25°	15°	8	4
12	30	Male	Left	20°	0°	0°	4	3
13	26	Female	Right	25°	0°	0°	4	2.5
14	23	Male	Right	40°	0°	0°	6	4
15	20	Male	Right	30°	10°	10°	5	3
16	22	Female	Left	35°	15°	10°	4	2
Range	19–32			20°–45°	10°–25°	5°–15°	3–12	2–4
Mean ± SD	23.2 ± 4.2			32.8 ± 7.9	16.8 ± 5.1	10.6 ± 3.2	5.6 ± 2.3	2.8 ± 0.8

*LLD* limb-length discrepancy

Limb lengthening was performed in all cases through the distal femoral osteotomy to address the residual limb shortening. The mean external fixator time (the time spent in the external fixator) was 4.6 ± 0.97 months. The mean external fixator index (the time in external fixation divided by the lengthening in months/cm) was 1.6 ± 0.24.

The follow-up period ranged from 60 to 132 months (mean 85.6 ± 23.5). At the time of last follow-up, the HHS showed an excellent functional outcome in two cases (12.50 %), good in 13 cases (81.25 %) and fair in one case (6.25 %). There was no poor functional outcome. Preoperatively, the mean HHS was 56.18 ± 7.41 points, and at the time of last follow-up, it improved to a mean of 84.62 ± 4.19 points (Table 2). Improvement in the functional outcome was statistically significant (*p* ≤ 0.001). The functional outcome was noted to be significantly better:

**Tables 2:** Results of treatment

No.	Limb lengthening (cm)	External fixator time (months)	External fixator index	Harris hip score		Follow-up (months)
				Preoperative	Postoperative	
1	2.5	4	1.6	50	85	124
2	4	6	1.5	45	85	132
3	3	4	1.3	60	88	100
4	2	3.5	1.7	65	90	64
5	2	4	2	55	84	72
6	4	6	1.5	68	88	100
7	2.5	3.5	1.4	65	88	64
8	2	4	2	60	85	70
9	2	4	2	56	80	86
10	3	5	1.6	50	84	66
11	4	6	1.5	45	75	122
12	3	4	1.3	60	92	70
13	2.5	5	2	60	85	78
14	4	6	1.5	45	80	90
15	3	5	1.6	60	82	60
16	2	3.5	1.7	55	83	72
Range	2–4	3.5–6	1.3–2	45–68	75–92	60–132
Mean ± SD	2.8 ± 0.79	4.6 ± 0.97	1.6 ± 0.24	56.18 ± 7.41	84.62 ± 4.19	85.6 ± 23.5

1. in male than female patients;
2. when the apparent LLD was less than 6 cm;
3. when the distal femoral lengthening was less than 3 cm; and
4. when the external fixator time was 4 months maximally (Table 3).

**Table 3:** Factors affecting clinical outcome

Factors		Harris hip score				Statistical analysis	
Group	Subgroup	Excellent	Good	Fair	Total	Statistical value	*p* value
Age groups	18–20	1	5	0	6	4.433*	0.066
	21–30	1	7	1	9		
	31–40	0	1	0	1		
Gender	Males	2	7	0	9	3.603**	0.2
	Females	0	6	1	7		
Initial LLD	Less than 6 cm	2	8	0	10	3.167*	0.115
	6 –10 cm	0	4	1	5		
	More than 10 cm	0	1	0	1		
Distal femoral lengthening	Less than 3 cm	1	7	0	8	4.194**	0.2
	3 cm or more	1	6	1	8		
External fixator time	Up to 4 months	2	7	0	9	3.603**	0.2
	More than 4 months	0	6	1	7		

*LLD* limb-length discrepancy

* *F* value of one-way analysis of variance test

** *H* value of Kruskal–Wallis test

Pain subsided in all patients and the Trendelenburg sign became negative in all but three (19 %) patients. None had a limb-length discrepancy (as measured clinically by the block method), and the alignment of the extremity was reestablished in all cases.

Superficial pin tract infections occurred in all cases and were managed by frequent pin site care and oral antibiotics. No cases of deep infection or neurovascular injury occurred as a result of this treatment. At the time of last follow-up, three patients complained of lurch while walking; however, no additional procedures were required. No cases of regenerate fracture were encountered in this study.

## Discussion

A resection arthroplasty of the hip joint (Girdlestone) has been described for alleviation of pain and improvement in hip function, but this arthroplasty produces an unstable joint, limb-length discrepancy and functional disability [10]. The pelvic support osteotomy (PSO) was introduced to improve hip stability by directly transferring the body weight to the distal femur and to relieve pain by offloading the stump of the femoral head [6, 11, 12]. The aim of the PSO is to support the pelvis on the femur, reduce lumbar lordosis and increase the distance of the greater trochanter from the pelvis, which in turn tensions the gluteus medius muscle and reduces the Trendelenburg limp [7, 8]. Milch described a similar angulation osteotomy with the addition of femoral head resection in patients with anterior dislocation who had developed arthrosis [6]. A shortcoming of the original PSO is the abducted position of the lower limb places high stresses on the knee joint, and it does not address the limb-length discrepancy [8].

Ilizarov added another distal osteotomy to restore the orientation of the knee and ankle joint lines in the coronal plane and to allow femoral lengthening while maintaining the advantages of the proximal osteotomy in lateralizing and displacing the greater trochanter and, in so doing, increasing the efficiency of the abductor muscles [8]. A successful Ilizarov hip reconstruction (IHR) reduces limp through abolishing the Trendelenburg lurch (due to elimination of any further adduction between the femur and the pelvis which then prevents pelvic drop during the single stance phase of gait), equalizes limb length and facilitates a more energy-efficient gait through the stability provided to the hemipelvis [7, 11–17]. In Ilizarov hip reconstruction, the mechanical axis of the lower limb should, ideally, go upward as an extension of the normal mechanical axis of the tibia (from the center of the ankle to the center of the knee joint) through the original acetabulum perpendicular to the horizontal line of the pelvis (line connecting top of both iliac crests). The proximal part of the femur should lie parallel to the lateral wall of the pelvis so that in performing the Trendelenburg test, the pelvis rests on the proximal femur with zero-degree adduction and so produces the negative Trendelenburg sign.

It is recommended that the hip joint should be mobile or the femoral head absent for a successful PSO. In this cohort, we performed a resection of the femoral head remnants to produce mobilization of the hip joint; this procedure also served to correct the fixed contractures of the hip joint and reduce that component of limb shortening arising from joint contractures. This increased range of movement then allowed for maximum adduction of the limb prior to performing the more proximal osteotomy.

Hip pain was present in all patients in this cohort. This had reduced postoperatively. The mean preoperative limb-length discrepancy of 5.6 ± 2.3 cm (range 3–12) was reduced substantially after hip resection and mobilization and required a reestimation of the required limb lengthening. In all patients, the limb-length discrepancy was corrected through the distal osteotomy. Due to the muscle bulk of the thigh, the distal femoral osteotomy, which included a varus correction, did not produce a clinically visible deformity.

The HHS improved significantly compared with the preoperative values. Several other studies have confirmed the beneficial effects of the Ilizarov hip reconstruction using clinical and imaging studies and through gait analysis. The improvement in HHS after IHR has been attributed to the increased hip abductor muscle mass after PSO (documented with MRI studies) and the decreased joint reaction forces (as confirmed by gait analysis parameters) [7, 13, 17, 18].

In a biomechanical study, Inan et al. [19] showed a distal and lateral translation osteotomy of the greater trochanter after a traditional PSO increases the length of the abductor moment arm more than that obtained by traditional PSO alone. In our study, a residual lurching gait was present in three cases (18.7 %) and was clinically significant in one patient (6.2 %). This positive Trendelenburg sign may be attributed to: (1) weakness of hip abductors due to long-standing disuse; (2) pressure atrophy of the tissues interposed between the angulated proximal femur and the lateral wall of the pelvis which subsequently permits some of adduction and a return of the positive Trendelenburg sign. Some authors recommend an overcorrection of the proximal osteotomy (about 15° of additional valgus) in anticipation of this loss of support in maximum adduction, but it is our opinion that there may be some unpredictability in the functional results and patient’s satisfaction after this increased valgus angulation.

The main adverse effect of Ilizarov hip reconstruction osteotomy is the altered anatomy of the proximal femur in both sagittal and frontal planes which would increase the difficulty of a future total hip replacement (THR). However, Thabet et al. [5] have reported a successful THR 15 years after a PSO and concluded that the altered anatomy of the proximal femur after PSO did not preclude the subsequent THR; however, the THR can be tricky and careful attention to the surgical details is necessary to achieve a successful outcome.

In the light of these midterm results, we conclude that the Ilizarov hip reconstruction is a valuable and durable solution for the late sequelae of childhood septic arthritis of the hip.
